# How Macrophages Become Transcriptionally Dysregulated: A Hidden Impact of Antitumor Therapy

**DOI:** 10.3390/ijms22052662

**Published:** 2021-03-06

**Authors:** Galina F. Medvedeva, Daria O. Kuzmina, Julia Nuzhina, Alexander A. Shtil, Marina S. Dukhinova

**Affiliations:** 1 International Institute ‘Solution Chemistry of Advanced Materials and Technologies’, ITMO University, 191002 Saint-Petersburg, Russia; medvedeva@scamt-itmo.ru (G.F.M.); kuzmina@scamt-itmo.ru (D.O.K.); shtil@scamt-itmo.ru (A.A.S.); 2 Institute of Gene Biology, Russian Academy of Science, 119334 Moscow, Russia; julia.nuzhina@gmail.com

**Keywords:** tumor-associated macrophages, transcription factors, cancer, immunotherapy, radiotherapy, chemotherapy, oncoimmunology

## Abstract

Tumor-associated macrophages (TAMs) are the essential components of the tumor microenvironment. TAMs originate from blood monocytes and undergo pro- or anti-inflammatory polarization during their life span within the tumor. The balance between macrophage functional populations and the efficacy of their antitumor activities rely on the transcription factors such as STAT1, NF-κB, IRF, and others. These molecular tools are of primary importance, as they contribute to the tumor adaptations and resistance to radio- and chemotherapy and can become important biomarkers for theranostics. Herein, we describe the major transcriptional mechanisms specific for TAM, as well as how radio- and chemotherapy can impact gene transcription and functionality of macrophages, and what are the consequences of the TAM-tumor cooperation.

## 1. Introduction

Tumor-associated macrophages (TAMs) are essential components of the tumor microenvironment, along with other immune cells, fibroblasts, and neovasculature [1,2]. Macrophages, including TAMs, interact with the surrounding milieu and exhibit functional diversity with the specific release of pro- and anti-inflammatory cytokines and growth factors [3,4,5,6,7,8]. TAMs represent a mixed cell population, which includes pro- and anti-inflammatory activated macrophages and newly infiltrated macrophages and monocytes that migrate from the surrounding tissues or through the blood vessel wall, respectively, and undergo further alterations in the tumor microenvironment. Of interest, phenotypically and functionally, TAMs are more similar to tissue-resident cells, such as alveolar macrophages (lungs), Kupffer cells (liver), microglia (the central nervous system), and others, depending on the tumor location, but not to blood-derived monocytes.

Generally, macrophages are classified as resting (non-activated, M0), proinflammatory (classically activated, M1), or anti-inflammatory (alternatively activated, M2) [9]. Tumor microenvironment contains a plethora of signaling and chemoattractant molecules, which flexibly balance the monocyte/macrophage pro- or anti-inflammatory polarization (Table 1 and Table 2). M1 activation is induced in response to Toll-like receptor (TLR) ligands such as bacterial lipopolysaccharide (LPS) and lipoteichoic acid, or proinflammatory cytokines such as tumor necrosis factor alpha (TNF-α) and interferon gamma (IFN-γ) [10]. The major M1-associated transcriptional patterns require the participation of nuclear factor kappa-light-chain-enhancer of activated B cells (NF-κB) and signal transducer and activator of transcription 1 (STAT1) in cooperation with interferon regulatory factor (IRF) 9, p53, and other transcription factors (TFs) (Table 1) [11,12]. M1-like macrophages show a high capacity for antigen presentation and increased production of nitric oxide (NO), reactive oxygen species (ROS), and proinflammatory cytokines (interleukin (IL) IL-1β, IL-6, tumor necrosis factor alpha (TNF-α), and others), playing a dual role in the tumor microenvironment [13,14]. Proinflammatory activity improves the therapeutic outcome in patients with breast, ovarian, lung, and other types of cancer [3,4,5]. At the same time, M1-like macrophages stimulate cancer cell motility and are associated with tumor progression in certain cases, such as pancreatic and gastric cancer [8,15,16]. Cytokines, such as IL-4, IL-6, IL-10, IL-13, chemokine (C-C motif) ligand (CCL) 2, TNF-α, transforming growth factor beta (TGF-β), or prostaglandin E2 (PGE2), functionalize anti-inflammatory macrophage polarization, thereby suppressing immunity and stimulating angiogenesis, tissue remodeling/repair, and self-antigen tolerance [17,18,19,20,21]. Anti-inflammatory polarization requires activation of STAT3, STAT6, and NF-κB p50-p50 homodimer (Table 2) [22,23]. M2-like cells release cytokines IL-4, IL-10, IL-13, and growth factors TGF-β, vascular endothelial growth factor (VEGF), epidermal growth factor (EGF), which exhibit pro-tumorigenic properties: stimulate cell proliferation, tumor encapsulation, and vasculogenesis and promote drug resistance [6,7].

Additionally, the M1/M2 ratio and the exact cytokine secretion profile of macrophages vary with respect to cancer stage and tumor microenvironment [53,54]. With the disease progression, the number of M2-like TAMs gradually increases in the tumor microenvironment due to its immunosuppressive activities and abnormal blood vessel fenestration, as it is shown for lung carcinoma, cutaneous melanoma, colorectal, prostate, and ovarian cancer [55,56,57,58,59]. Moreover, the transition from acute to chronic inflammation, which occurs at the late stages, reduces antitumor immunoreactivity and increases growth factor production with subsequent tumor growth and metastasis [60,61].

**Figure 1:**
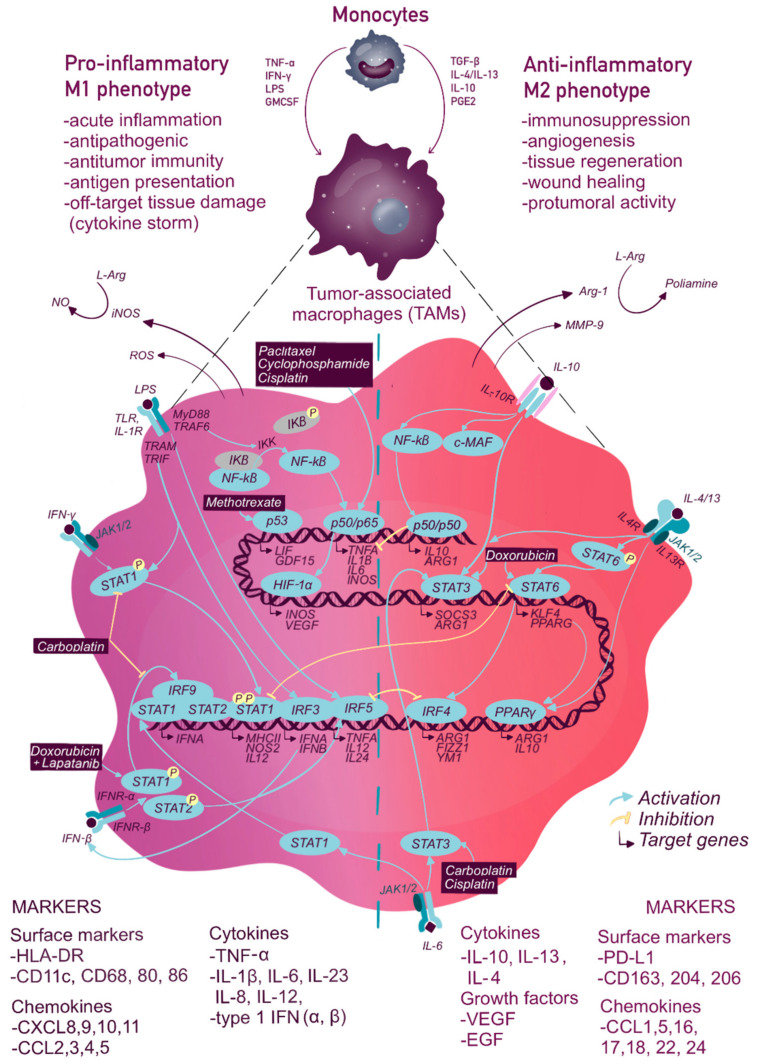
Transcription factors involved in macrophage polarization: impact of tumor microenvironment and chemotherapy. The activation or inhibition of signaling pathways are indicated by the blue arrows or by the yellow suppression symbols, respectively. The principal chemotherapeutic drugs, which impact macrophage transcriptional profiles, are shown. TAMs transcriptional patterns require phosphorylation (P) and activation of NF-κB, STATs, IRFs, p53, and other TFs (Table 1) [11,12]. NF-κB (P65-p50 heterodimer) becomes activated upon LPS stimulation and acts as a positive regulator of proinflammatory gene transcription in macrophages [28,45,64,65,66]. However, anti-inflammatory macrophage activity is also NF-κB-dependent [64,67]. P50-p50 homodimers inhibit proinflammatory genes. The roles of NF-κB in M1/M2 balance are likely associated with p65/p50 ratio [68,69,70]. Hypoxia-inducible factor 1-alpha (HIF-1α) and p53 are also associated with the proinflammatory macrophage phenotype [24,52,71,72,73,74]. IRF3 and 5 are involved in proinflammatory macrophage polarization following IFN-α/β (interferon alfa/beta) and IFN-γ receptor engagement while IRF4 is involved in IL-4-induced macrophage polarization [65,75,76]. TLR and IFN-γ receptor signaling induces macrophage proinflammatory activity with STAT1-dependent increase in *NOS2, IL12*, MHC (major histocompatibility complex) class II expression and enhanced antigen presentation [31]. IL-4 and IL-13 induce alternative polarization implying TFs STAT3 and STAT6 [77,78,79,80]. M1-like macrophages show increased production of NO, ROS, and proinflammatory cytokines IL-1β, IL-6, TNF-α, and others [13,14]. M2-like cells release cytokines IL-4, IL-10, IL-13, and growth factors TGF-β, VEGF, EGF that exhibit pro-tumorigenic properties [6,7]. Chemotherapeutic agents such as paclitaxel, cyclophosphamide, and cisplatin induce M1 polarization via NF-κB (p50/p65) [4,81,82,83,84]. Cisplatin and carboplatin alter M2 differentiation via STAT3, while carboplatin suppresses STAT1 and STAT6 [85,86]. Doxorubicin in combination with lapatinib activates immature macrophages via STAT1 [87].

The pro- or anti-oncogenic function of macrophages is precisely controlled by the specific TFs, which can be used as additional diagnostic and immunotherapeutic tools (Figure 1) [13,62,63].

TF activities depend on the disease stage and therapeutic regimen. However, transcriptional programming of macrophages during chemo- and radiotherapy is not fully investigated. In this review, we focus on the role of TAMs in cancer therapy. We address the transcriptional mechanisms of macrophage polarization in response to chemotherapy or ionizing radiation and the routes for macrophages-supported immunotherapy to improve the outcome of the disease.

## 2. Macrophage Transcriptional Reprogramming during Chemo- and Radiotherapy

Tumor resistance relies directly on the interaction between cancer cells and their surrounding [6,88]. TAMs are among the major tumor microenvironment components, which significantly restrict therapeutic efficiency and mediate tumor resistance via several essential mechanisms. First, macrophages stimulate drug metabolism and removal from the cancer cell [89]. Second, tumorigenic cytokines, such as IL-6, insulin-like growth factors (IGF) 1 and 2, produced by TAMs, also promote drug resistance in various cancer types [90,91,92]. Finally, M2-like macrophages tend to accumulate in proximity to the tumor blood vessels following therapy and release the plethora of growth factors, leading to revascularization and tumor relapse [93]. Given that, the TAM functionality is a major factor that determines the treatment outcome (Table 3).

**Figure 2:**
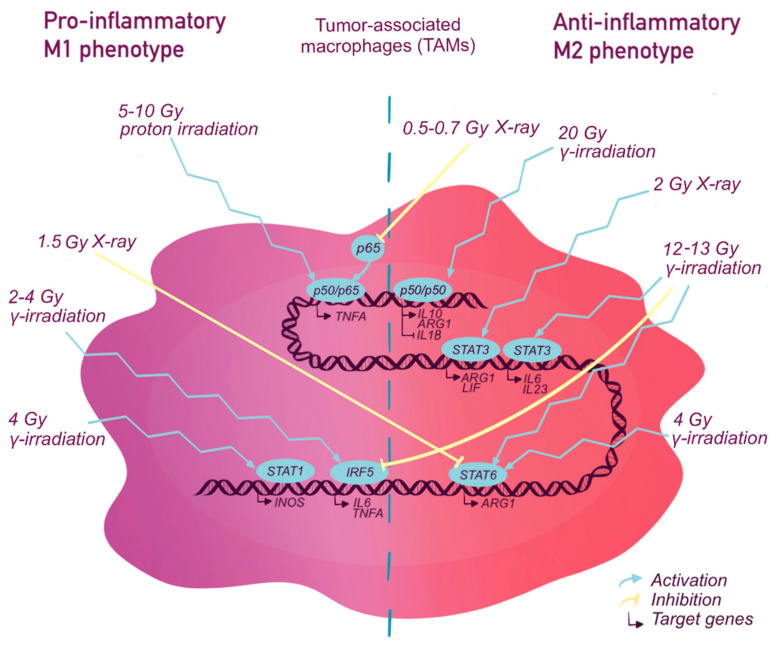
Transcriptional activity of tumor-associated macrophages during radiotherapy. The activation or inhibition of signaling pathways are indicated by the blue arrows or by the yellow suppression symbols, respectively. Gy-Gray (Units). The most common types of radiotherapy applied in clinics are gamma (γ)-, proton-, and X-ray irradiation (Figure 2). Irradiation doses range from low (less than 1 Gy) and moderate (2–10 Gy) to high (>10 Gy) and can be applied as a single course or fractionated [169,170,171]. G -irradiation (2 Gy) induces M1 phenotype via IRF5 activation [145]. Bigger doses (>12 Gy) induce M2 phenotype via STAT3 and STAT6 activation with IRF5 suppression and p50-p50 activation, respectively [149,150,151,153]. Four Gy 𝛾-irradiation activates STAT1 and STAT6, up-regulates M1 and M2 markers [172]. 1.5 Gy X-ray induces macrophage polarization toward M1 phenotype via STAT6 suppression while 5–10 Gy proton irradiation induces M1 phenotype via p50-p65 nuclear translocation [68,156]. Two or eight Gy X-ray induce M2 phenotype via STAT3 activation while 0.5–0.7 Gy X-ray down-regulates M1 markers via inhibition of p65 nuclear transport [158,160,173].

While therapeutic responses are modulated by TAMs, chemo- and radiotherapy also show direct and indirect effects on macrophage survival and activity. The direct action is associated with the off-target cytotoxicity and pro- or anti-inflammatory activation of macrophages. The majority of TAMs are polarized to M2-like phenotype and can release numerous tumor-promoting factors before the treatment. Radio- or chemotherapy can prevent these undesired events reducing M2 macrophage numbers and attracting the blood-derived monocytes with the higher anti-tumor potential. The monocyte infiltration and proinflammatory signaling are supported via pro-inflammatory cytokines and chemokines (ex., CCL2) from the tumor microenvironment (Figure 1 and Figure 2) increased blood vessel wall permeability and signaling from dead and damaged cancer cells [87,162].

The indirect mechanisms are caused by the incoming signals from (a) the damaged, dying, and, at the later time points, resistant cancer cells and (b) the altered tumor microenvironment. Upon chemical and irradiation exposure, surviving cancer cells produce pro-tumor cytokines as IL-17, stromal cell-derived factor-1 (SDF-1), CCL2, and colony-stimulating factor 1 (CSF-1), which limit the therapeutic benefits [44,163,164,165]. At the same time, cancer cell damage and ROS generation induce proinflammatory macrophage activities, as phagocytosis, antigen presentation, and production of proinflammatory factors such as inducible nitric oxide synthase (iNOS) and TNF-α, and lymphocyte chemoattraction [85,124,125,166,167,168]. With that, the therapeutic impact on TAMs remains controversial with possible pro- or anti-inflammatory polarization and hardly predictable outcomes. As macrophage polarization and cytokine production rely on the cell transcriptional machinery, we aim to discuss the major TFs involved in cytokine network regulation-NF-κB, STAT-family, IRF-family, and p53,-in the context of the tumor microenvironment and chemo- and radiotherapy (Figure 1 and Figure 2, Table 3).

### 2.1. The Nuclear Factor Kappa B (NF-κb)

The NF-κB/Rel TF family in mammals consists of five proteins-p65 (Rel-A, transcription factor p65), p50 (NF-κB1, nuclear factor NF-kappa-B p105 subunit), p52 (NF-κB2, nuclear factor NF-kappa-B p100 subunit), c-Rel (proto-oncogene c-Rel), and RelB (transcription factor RelB), with possible 15 combinations of homo- and heterodimeric complexes. All of these proteins contain a characteristic N-terminal Rel homology domain (RHD) required for dimerization and target DNA binding [174]. NF-κB participates in cell metabolism and is also involved in responses to external stimuli such as cytokines, ROS, heavy metals, irradiation, bacterial, and viral infection. Additionally, NF-κB is a well-known mediator of tumor-associated inflammation [175,176].

The reported roles for NF-κB in TAMs are associated with both pro- and anti-inflammatory functions [64]. It is well-known that NF-κB transcription is driven upon LPS stimulation and results in proinflammatory cytokine production (Table 1) [28,45,65,66]. NF-κB signaling is inhibited in M2-like TAMs in many tumors including glioblastoma, ovarian cancer, hepatocellular carcinoma, and others [176,177]. At the same time, macrophage anti-inflammatory activity is also controlled by NF-κB [64,67]. NF-κB p50 and p65 subunits are both embedded into M2-like polarization with high IL-10 and low IL-12 secretion profiles [44,178,179,180]. The roles of NF-κB in M1/M2 balance are likely associated with a p65/p50 ratio with p65-p50 heterodimers supporting proinflammatory functions and p50 homodimers acting as anti-inflammatory mediators [68,69,70]. NF-κB functionality is essential for TAM transcriptional reprogramming and prediction of the outcome for chemo-, radio, and immunotherapy.

#### 2.1.1. NF-κB and Chemotherapy

Numerous drugs impact NF-κB transcriptional activity guiding TAMs toward M1 polarization. Taxol, cyclophosphamide, and cisplatin stimulate NF-κB p50/RelA and p50/c-Rel subunits and upregulate proinflammatory cytokine (*TNFA, IL12, INOS, COX2*) and *TLR* gene expression while downregulating anti-inflammatory *IL10* and *TGFB* (Figure 1) [82,83,84,107]. Interestingly, combinations of cyclophosphamide, vincristine, and doxorubicin with immunotherapy induce only partial M1-activation of TAMs [81].

NF-κB plays a critical role not only in macrophage polarization but also in their metabolism and surveillance in the tumor microenvironment upon chemotherapy. Such effects of chemotherapy are mediated via caspase-8-dependent apoptosis, which is selectively activated in monocytes and tumor-associated phagocytes upon trabectedin treatment and in M2-like TAMs upon platinum-containing drug exposure) [85,181,182,183,184]. Upon chemotherapy, NF-κB acts as an important proinflammatory inductor and regulator of macrophage viability.

#### 2.1.2. NF-κB and Radiotherapy

Radiotherapy can shift the balance between NF-κB subunits depending on the irradiation dose (Figure 2). Low radiation doses increase the nuclear translocation of p50-p50 homodimer and inhibit p65 translocation, thereby reducing IL1B expression and proinflammatory macrophage activity [173]. Moderate doses (5–10 Gy) preferentially stimulate p65-p50 transcriptional activity in macrophage thereby reprogramming them into M1 phenotype with increased TNF-α, IL-6, IL-8 and reduced EGF [68]. High irradiation dose utilizes p50 subunit for M2 polarization and maintenance of the immunosuppressive microenvironment following radiotherapy [153]. NF-κB mediates pro-survival signaling in macrophages exposed to 10 Gy and higher cumulative doses [148]. This mechanism partially protects macrophages from irradiation. The preferred strategy for NF-κB implementation in radiotherapy is to target p50/p65 and to consider the doses of irradiation.

### 2.2. STAT Transcription Factor Family

The STAT family consists of 7 TFs including STAT1, STAT3, STAT6, which play an essential role in macrophage polarization. TLR and IFN-γ receptor signaling induces macrophage proinflammatory activity with STAT1-dependent increase in *NOS2, IL12,* MHC (major histocompatibility complex) class II expression and enhanced antigen presentation [31]. IL-4 and IL-13 induce alternative polarization with high levels of IL-10 and Arg-1 (Arginase-1), implying TFs STAT3 and STAT6 [77,78,79,80]. STAT3 can also orchestrate NF-κB-mediated transcription and promote pro-tumor chronic inflammation [33,185,186,187].

#### 2.2.1. STATs and Chemotherapy

Chemotherapy has a strong impact on macrophage STAT activities resulting in TAM abundance and phenotypic alterations. Among the routinely applied anticancer drugs, cisplatin and carboplatin increase STAT3 and STAT6 activity and M2-like phenotype of the TAMs [85]. Doxorubicin treatment alone stimulates STAT6 and is also associated with anti-inflammatory effects. At the same time, combined therapy with doxorubicin and cyclophosphamide or EGFR inhibitor lapatinib implies M1-associated STAT1 to stimulate and prolong macrophage antitumor activity [87]. Imatinib and paclitaxel inhibit the STAT6 pathway and M2-like cytokine production in macrophages showing, thus, proinflammatory potential [4,188]. Taken together, STAT TFs exhibit multidirectional effects in TAM functionality due to counterplay between the various STAT members, NF-κB, and other TFs.

#### 2.2.2. STATs and Radiotherapy

Various radiotherapeutic strategies have a differential and, sometimes, non-specific impact on the members of the STAT TF family [172]. The generalized effect of X-ray and 𝛾-photon radiotherapy is STAT3 activation, which is observed in TAMs in response to all clinical doses of radiation. Irradiation promotes IL-6 production by the tumor microenvironment, which results in STAT3 phosphorylation and subsequent anti-inflammatory CCL2, CCL4, VEGF, and TGF-β cytokine production [149,158,160,189]. It is worth noting that STAT3 signaling also promotes cell survival after irradiation exposure via induction of anti-apoptotic proteins (survivin and Bcl-2), and this effect is more profound for M2-like TAMs [158,160,190]. The low radiation doses show a bidirectional impact on the anti-inflammatory TFs, comprised of STAT3 stimulation, as mentioned earlier, and STAT6 suppression with high IL-5 and 13 and low TGF-β cytokine profile [150,156]. Considering that NF-κB is also suppressed upon low-dose radiotherapy, macrophages may finally acquire anti-inflammatory characteristics, although this has to be further studied. Intermediate radiation doses stimulate the transcriptional activities of STAT1, STAT3, and STAT6 [172]. The immunomodulatory effect of such treatment is the most difficult to control, as it simultaneously triggers intracellular pro- and anti-inflammatory signaling pathways. The functional outcome likely depends on individual cell characteristics (time after infiltration of the tumor), the other TFs impacted by radiotherapy (for example, NF-κB) and tumor microenvironment [191]. Finally, high-dose radiation activates STAT6 and therefore has the most pronounced anti-inflammatory effect [149,156].

### 2.3. Interferon Regulatory Factor (IRF)

The IRF family is represented by nine members ranging from IRF1 to IRF9. IRFs promote host defense against viral and microbial pathogens by regulating type I and II IFN-responsive genes. IRF TFs are also active in TAMs and are linked to the pro- and anti-inflammatory cytokine production [192]. IRF3, 5, 7, and 8 are involved in proinflammatory macrophage polarization and control of chemokine (S100A8, S100A9S100a9, matrix metallopeptidase (MMP) 9 and 14, CXCL2, and CCL5) production [65,75,193]. IRF3, along with IRF4, can also exhibit an alternative activity inducing the expression of anti-inflammatory genes *IL1RA, IL10, IFNB* [76,194].

In the context of carcinogenesis, IRFs support tumor-driven macrophage activity, particularly, promoting T-cell exhaustion, while also controlling neovascularization and proinflammatory features of TAMs [39,42,75,195,196,197,198,199]. It is also worth noting that IRFs are commonly involved in STAT-mediated transcription and have to be considered as a complex transcriptional network [43,45,59,71].

#### 2.3.1. IRFs and Chemotherapy

IRF TFs strongly echo the STAT-mediated transcription in macrophages and it becomes challenging to evaluate exclusively IRFs contribution to macrophage function. The majority of data show that chemotherapy-induced IRF activation supports the tumoricidal activity of macrophages. For instance, IRF5 activation and, subsequently, macrophage proinflammatory functionalization are induced by PARP inhibitor olaparib [145]. However, some studies show that IRFs are involved in M2-like polarization following chemotherapy and can be associated with resistance [200,201].

#### 2.3.2. IRFs and Radiotherapy

The IRF input into macrophage polarization upon radiotherapy remains poorly investigated. In fact, according to the authors’ knowledge, alterations in IRF1, 2, 3, and 5 activity were reported in irradiated macrophages so far [72,73,202]. Low dose-irradiation induces ROS generation with subsequent ATM activation to stimulate IRF5 expression and M1-like macrophage polarization with increased IL-6, TNF-α, and IFN-γ [145]. Furthermore, IRF5 cooperates with the NF-κB Rel-A subunit, regulates proinflammatory cytokine gene expression, which can result in M1 polarization. However, the IRF5-NF-κB interactions have not yet been studied in irradiated macrophages [71]. Moderate dose-radiotherapy stimulates IRF1 and 5 revealing the potential proinflammatory effects. High radiation doses reduce IRF2 and 5 levels increasing numbers of M2-like macrophages in the tumor [151]. At least for the high-dose irradiation IRF1 is suppressed in monocytes, while upregulated in macrophages, suggesting the different responses from infiltrated cells and TAMs [72,73].

### 2.4. P53

Despite the extensive studies on the tumor suppressor p53, its roles in immune cells, including TAMs, are not fully understood. It is known that p53 regulates inflammatory responses in the tumor microenvironment. In macrophages p53 is involved in cell survival and death, monocyte-to-macrophage differentiation, and M1/M2 polarization [52,74,203,204]. P53 is up-regulated and activated following both pro- and anti-inflammatory macrophage activation and promotes pro-apoptotic pathways predominantly in M1 cells [205,206].

The majority of observations suggest that p53 is required for anti-tumor activities of TAMs, as p53 is generally associated with the proinflammatory macrophage phenotype, increased levels of IL-6 and IL-12, and enhanced phagocytic activity [52,74,203,204,207]. At the same time, p53 may restrict the performance of other M1-associated TFs STAT1, NF-κB, IRF9, IRF5, and c-Myc [52,204,208,209,210]. In accordance, the p53 transcriptional activities and high levels of its target genes *CDKN1A* (cyclin-dependent kinase inhibitor 1), *MDM2*, *PUMA*, and *BAX* are detected in TAMs along with increased *IL6* and *CXCL1* [211]. The outcome of p53-mediated transcription in the tumor microenvironment depends on the cooperation with the other signaling molecules, such as NF-κB, STAT1, and c-Myc, p21 and some others [52,203,208,209,210,212,213,214].

#### 2.4.1. P53 and Chemotherapy

P53 is involved in cell responses to cytotoxic, cytostatic, and targeted drugs, which impact macrophages. Doxorubicin, methotrexate 5-fluorouracil, and other chemotherapeutic compounds induce p53 activity in TAMs with subsequent increase in p53 target p21 and IL6 expression. The p53-driven proinflammatory polarization results in tumor sensitization to chemotherapy and is specific for monocyte/macrophage subsets [140]. Among the targeted drugs, nutlin-3, which inhibits p53/MDM2 interaction and stabilizes p53, MDM2 inhibitor APG-115, and anti-VEGF receptor 3 activate p53 and promote p53-NF-κB cooperation to stimulate antitumor macrophage reactivity [52,210,211,215]. P53 maintains the physiological levels of programmed cell death protein 1 (PD-1) ligand, restricts excessive extracellular vesicle formation, and subsequently limits T-cell exhaustion and immunosuppression. [216,217]. With that, p53 can become a multifaceted target for immunotherapy.

#### 2.4.2. P53 and Radiotherapy

As a pro-apoptotic protein, p53 sensitizes cells to radiation, and this effect is also present in irradiated macrophages. P53 becomes activated in macrophages during radiotherapy and is associated with TAM polarization and modulation of cell survival. However, the impact of different irradiation doses has not been studied. At least the intermediate (4–5 Gy) irradiation increases p53 protein levels and its transcriptional activity together with the antitumor (TNF-α, FasL) and tumorigenic activities (MMP-2, MMP-9) of macrophages [218,219]. P53 is directly linked to macrophage survival, as TP53-/- macrophages are highly radioresistant due to reduced caspase-8 expression [220]. Interestingly, p53 proapoptotic activity is stronger in M1, than in M2, macrophages, which correlates with the improved radioresistance of M2 cells [221]. Thus, p53 may regulate the balance of pro- and anti-inflammatory macrophages in the irradiated tumor microenvironment [221,222]. P53 plays an important role in macrophages simultaneously regulating cell functional activities and survival during chemo- and radiotherapy. These facts have to be considered when applying p53-targeted therapy.

### 2.5. Other Transcription Factors Affected by Radio- and Chemotherapy

Transcriptional networks essential for macrophage polarization include a broad range of TFs, which are also involved in therapeutic responses. When summarizing the existing data it can be observed that various treatments tend to impact M2-associated TFs, such as nuclear factor erythroid 2-related factor 2 (Nrf2), peroxisome proliferator-activated receptor gamma (PPAR-γ), cAMP response element-binding protein (CREB), and some others, which may be due to the immunosuppressive properties of the tumor microenvironment. However, a substantial number of transcriptional mechanisms have not yet been studied in the context of chemo- and radiotherapy.

Nrf2 shifts macrophages toward anti-inflammatory polarization altering cytokine, growth factor and cell adhesion molecule profiles [223,224,225]. Nrf2 deficiency in myeloid cells is associated with the enhanced metastatic profile of the tumor and tumorigenic immune activity [226,227]. The Nrf2 activation orchestrated by p21, which is observed after γ-irradiation, is dose-dependent, reduces oxidative stress in macrophages, and may, thus, protect the cells against γ-ray damage [228]. While the Nrf2 activities in chemotherapy have not yet been studied, Nrf2 controls local tissue inflammation and can potentially protect TAMs from drug-induced oxidative damage [229,230].

Peroxisome proliferator-activated receptors (PPARs) comprise another family of anti-inflammatory TFs [231]. Macrophage-specific PPAR-γ impairs the chemotherapeutic efficiency [232]. During radiotherapy, PPAR-γ is involved in macrophage activation by irradiated cancer cells and immunogenic antitumor activity thereby reducing cancer progression and metastasis [233]. Thus, regulators of PPAR-γ may emerge as promising candidates in targeting both cancer cells and the tumor microenvironment [234].

Other candidate TFs, which can shape macrophage behavior, include cAMP response element-binding protein (CREB) and CCAAT-enhancer-binding proteins (C/EBPs) TF family [235,236,237,238]. The phosphorylated CREB inhibits NF-κB activation, thus limiting proinflammatory responses [239,240]. Furthermore, overexpression of CREB in committed macrophages provokes myeloproliferative diseases [241]. The C/EBP family consists of six members, from C/EBP-α to C/EBP-ζ, which may function in opposite ways. For instance, C/EBP-α and C/EBP-δ are one of the most important TFs that contribute to the M1 polarization, whereas C/EBPβ is associated with M2 macrophage polarization [11,242]. Furthermore, C/EBPβ activated by vitamin D3 or its derivatives can aggravate alternative macrophage polarization [225,243]. Interestingly, C/EBPβ is downstream of the rapamycin kinase (mTOR) pathway which is a target of immunosuppressive and anticancer drugs [244,245]. Therefore, mTOR targeting drugs should be considered in the framework of macrophage polarization and could represent a novel therapeutic approach. High-mobility group box protein 1 (HMGB1) is a chromatin-binding factor that promotes M2 polarization by activation of the receptor for advanced glycation end-products (RAGE) [246]. High expression of HMGB1 in TAMs has been shown to enhance lymphatic metastasis [247]. Current observations show opposite effects after radiotherapy; HMGB1 release can lead to immunosuppression and potentiate macrophages reprogramming towards M1 phenotype [166,248]. Chemotherapy stimulates HMGB1 production to promote antigen-presenting function of immune cells [249].

Many other factors that have an impact on TAM polarization have been not yet sufficiently investigated. For example, the Maf family consists of a wide number of TFs including V-maf musculoaponeurotic fibrosarcoma oncogene homolog B (Mafb) and c-Maf. Both of them are highly expressed in TAMs and correlate with anti-inflammatory responses in both human and murine models [250,251,252]. At the same time, JunB governs functional behaviour of both pro and anti-inflammatory macrophages. JunB upregulates IL-1β in proinflammatory polarized macrophages while enhancing anti-inflammatory markers in M2-like cells [253]. MAP kinase-interacting kinase (Mnk2) promotes education of M2 phenotype through induction of anti-inflammatory marker translation by activation of eukaryotic translation initiation factor (eIF4E) [254]. All of them are promising research targets in the framework of resistance overcoming.

## 3. Macrophage Transcription Factors in Antitumor Therapy

Numerous preclinical and clinical studies confirm the promising effects of multitargeted combinations based on radio-, chemo-, and immunotherapy in cancer medicine [255,256]. The most prominent treatment effects on monocyte/macrophage subsets are associated with the increased immune cell recruitment to the tumor site and promoted antigen presentation, which are now recognized as essential components of the tumor microenvironment [257].

While numerous TAM signaling pathways become non-specifically activated by the drug or irradiation, immunotherapy can potentially direct macrophage polarization with enhanced antitumor activity [258]. In the current clinical practice the most common TAM immunotherapeutic targets are membrane receptors such as cluster of differentiation 40 (CD40) [259], SIRP-α [260,261], CXCR4 [262], C-C chemokine receptor 2 (CCR2), [263], CSF1R [264,265,266], which promote M1-like macrophage activation with enhanced antigen presentation activity and immune cell chemoattraction in lung, breast and colon cancer, Burkitt’s lymphoma, and leukemia [258,267,268,269]. The corresponding transcriptional networks have to be additionally considered in future theranostics [270,271,272].

TF-based macrophage regulation can be considered as one of the most potent and efficient strategies against tumor therapeutic resistance. As described previously, a substantial number of chemical agents are already available for TAM transcriptional reprogramming. For NF-κB-based approaches target deletion of IκB kinase β (IKKβ) or inhibition of downstream PI3K significantly improve antitumor activities [273,274]. TLR agonists can be used for stimulation of NF-κB-mediated transcription, as shown for alveolar macrophages and monocytes (paraquat), for lung, breast, and melanoma cancers (chloroquine), for melanoma and squamous cell carcinoma (imiquimod) [275,276,277]. Cancer-associated chronic inflammation may request the application of nonsteroidal-based anti-inflammatory drugs (NSAIDs), such as acetaminophen or sulindac, to suppress undesired NF-κB-promoted production of growth and metastatic factors. Furthermore, NF-κB inhibition can prevent cisplatin- and carboplatin-induced M2-like phenotype in cervical and ovarian cancer [85]. Other possible additives for oncotherapy may implement salicylates (aspirin, sulfasalazine, triflusal), antioxidants (pyrrolidine dithiocarbamate, N-acetylcysteine, vitamin E, vitamin C), and peptides (SN50, nuclear localization signal peptide, NEMO-binding domain peptide, Toll/interleukin-1 receptor domain-containing adaptor protein) for non-specific NF-κB regulation [278,279,280].

STAT family-based immunotherapy can enhance the effects of ɣ-radiation via regulation of macrophage subset and cytokine balance in the tumor microenvironment [281]. For instance, tyrosine kinase inhibitors (sunitinib, sorafenib), WP1066, plant-derived imodin (PM37), and resveratrol inhibit STAT TF activity in macrophages and restricts M2 polarization, suggesting the improved antitumor reactivity against pancreatic adenocarcinoma, breast, and lung cancer [49,160,191,258,282,283,284]. Additionally, STATs, IRFs, NF-κB, and c-MYC mediated polarization can be regulated by microRNAs. MicroRNAs regulate gene expression through translation repression or mRNA degradation. It has been shown as a prospective macrophage-centered diagnostic and therapeutic strategy [22].

In conclusion, macrophage TFs are among the prospective diagnostic markers and therapeutic targets, which allow repolarize TAMs and impact tumor microenvironment preventing tumor resistance. In macrophage-based immunotherapy, various immunomodulators can be applied to optimize the chemo- or radiotherapeutic outcome and prolong the treatment benefits. Currently NF-κB, STAT, and IRF transcriptional machinery, which is implied in cancer and immune cell functionality, present the most substantial potential for cancer immunotherapy. Tumor status and disease type have to be considered for preferential pro- or anti-inflammatory alterations.

## Figures and Tables

**Table 1:** Major transcription factors involved in proinflammatory macrophage polarization.

Transcription Factor	Activating or Inhibitory Stimuli	Target Genes (Up/Down Regulated)	Reference
NF-κB	Hypoxia ↑	Up: *HIF1A, COX2*	[24]
	LPS ↑	Up: *ADM, HIF1A, COX2, INOS, TNFA, IL6*	[25,26,27]
	Fungal polysaccharide↑	Up: *INOS, IL6, TNFA, COX2*	[28]
	Mechanical stretch ↑	Up: *INOS, TNFA, IL1B, IL6* Down: *ARG1, CD206, TGFB1*	[29]
STAT1	IFN-γ ↑	Up: *GBP6, CXCL10, CIITA, IRF1, CXCL11, IFIT2*	[30,31]
	HDL ↑	Up: *IL12B*	[32]
STAT3	LPS ↑	Up: *IL8 and TNFA*	[33]
IRF1	GM-CSF + IFN-γ ↑	Up: *INOS*	[34]
	IFN-γ ↑ IFN-ɑ ↑	Up: *IL12B, IL6, TNFA* Down: *IL10*	[35,36]
IRF2	LPS ↑	Up: *IL12B, IL12Rb1, IFNG, IL1B, IL6* Down: *TNFA*	[37]
IRF3	LPS ↑	Up: *IFNB1*	[38]
IRF5	IFN-γ + LPS/GM-CSF ↑	Up: *IL12B, IL23* Down: *IL10*	[39]
IRF6	IL-4 ↓	Up: *ARG1, IL10, PPARG*	[40]
IRF7	IFN-α, LPS ↑	Down: *IL10*	[41,42]
IRF8	IFN-γ ↑	Up: *TNFA*	[43]

**Table 2:** Major transcription factors involved in anti-inflammatory macrophage polarization.

Transcription Factor	Activating or Inhibitory Stimuli	Target Genes (Up/Down Regulated)	Reference
NF-κB	IL-17 ↑	Up: *ARG1, FIZZ1, YM1, CD206, CD163*	[44]
	IL-10 ↑	Down: *IL12B*	[45]
	GDF-15 ↑	Down: *INOS, TNFA*	[46]
STAT3	IL-6 + LIF/ERK5 ↑ IL-6 ↑	Up: *ARG1, VEGFA,* *TGFB1 and IL10* Down: *IL12B, INOS, TNFA* Up: *ARG1, FIZZ1, IL10*	[47,48]
STAT6	IL-4, IL-13 ↑	Up: *FN1, CCL22*	[49]
STAT5	IL-6 ↑	Up: *PDCD1LG2, CYP19A1*	[50]
IRF4	LPS ↑	Down: *TNFA, IL12B*	[51]
p53	IL-4 ↓	Up: *MYC, ARG1, FIZZ1*	[52]

Legend and abbreviations used in Table 1 and Table 2. The major transcription factors involved in pro-(Table 1) and anti- (Table 2) inflammatory polarization. Transcription factors (column 1) become activated following specific external stimuli (column 2), regulate certain gene expression (column 3), and participate in macrophage functional polarization.↑-activating stimulus; ↓-inhibitory stimulus; up–regulated genes; down–down-regulated genes. NF-κB-nuclear factor kappa-light-chain-enhancer of activated B cells; STAT-signal transducer and activator of transcription; IRF-interferon regulatory factors; LPS-lipopolysaccharide; IFN-ɑ-interferon alfa; IFN-γ- interferon gamma; HDL-high-density lipoprotein; GM-CSF-granulocyte-macrophage colony-stimulating factor; IL-interleukin; GDF-15-growth differentiation factor 15; LIF-leukemia inhibitory factor; HIF1A-hypoxia-inducible factor 1-alpha; COX2-prostaglandin-endoperoxide synthase 2; ADM-adrenomedullin; INOS-inducible nitric oxide synthase; TNFA-tumor necrosis factor; ARG1-arginase-1; CD-cluster of differentiation; TGFB1-transforming growth factor beta; GBP6-guanylate binding protein; CXCL-C-X-C motif chemokine ligand; CIITA-class II major histocompatibility complex transactivator; IFIT2-interferon-induced protein with tetratricopeptide repeats 2; IFNB1-interferon beta 1; IFNG-interferon gamma; PPARG-peroxisome proliferator-activated receptor gamma; VEGFA-vascular endothelial growth factor A; FN1-fibronectin 1; CCL22-C-C motif chemokine ligand 22; PDCD1LG2-programmed cell death 1 ligand 2; CYP19A1-cytochrome P450 family 19 subfamily A member 1.

**Table 3:** Effects of chemotherapy and radiotherapy on macrophage phenotypes.

Therapeutic Intervention	Tumor Type	Mechanism of Action	Impact on Macrophages
Chemotherapy			
Doxorubicin	Breast, bladder carcinoma, Kaposi sarcoma, lymphoma, acute lymphocytic leukemia [94,95,96,97,98]	- Intercalates within DNA base pair; - Inhibits DNA and RNA synthesis; - Inhibits topoisomerase II causing DNA damage and induction of apoptosis [99]	- In combination with lapatinib: suppresses TAMs, activates immature macrophages via STAT1 and recruits them to the tumor site [87]; - In combination with cyclophosphamide, vincristine and immunotherapy induces M1 polarization [81]; - Induces M2 polarization via JAK2/STAT3 pathway, stimulates CD206, and decreases iNOS production [77]; - Induces M2 polarization via STAT6, up-regulates *IL10* and *TGFB1* expression [100]
Taxol (Paclitaxel)	Ovarian, breast, lung, sarcoma Kaposi, cervix, pancreas [101,102,103,104,105]	- Stimulates tubulin polymerization, anti-mitotic and proapoptotic activity [106]	- Induces M1 polarization via NF-κB (p50/RelA and p50/c-Rel) and IRF; - Stimulates TNF-α and IL-12 production [4,82]
Cyclophosphamide (CY)	Hodgkin, non-Hodgkin, cutaneous T-cell lymacrophageomas, multiple myeloma, leukemia, retinoblastoma, neuroblastoma, ovarian cancer, breast cancer [107,108,109,110,111,112,113,114]	- Alkylates DNA to trigger apoptosis; - Decreases the IFN-γ and IL-12 production; - Increases Th2 cytokine (IL-4, IL-10) levels in the cerebrospinal fluid and peripheral blood; - Shows immunosuppressive activity [115,116]	- Activates NF-κB; - Stimulates the production of proinflammatory IL-6 and IL-12 cytokines and decreases anti-inflammatory IL-10 and TGF-β production [83]; - Increases the number of M1 TAMs in a mouse model [81]
Cisplatin	Testicular, ovarian, cervical, breast, bladder, head and neck, esophageal, lung, mesothelioma, neuroblastoma [117,118,119,120,121,122]	- Interacts with DNA forming crosslink adducts; - Activates pro-apoptotic signaling pathways [123]	- Modulates M1 TAM phenotype and stimulates iNOS, TNF-α, IL-1β, IL-12, and IFN-γ production [124,125]; - Alters M2 differentiation via STAT3, stimulates IL-10, IL-12 production [85]
Carboplatin	Ovarian, lung, head and neck, endometrial, esophageal, bladder, breast, cervical cancers; central nervous system or germ cell tumors; osteogenic sarcoma [126,127,128,129,130,131,132]	- Forms reactive platinum complexes; - Forms the cross-linked adducts of DNA molecules; - inhibits DNA synthesis in all the phases of the cell cycle [133]	- Induces M2-like phenotype via STAT3 activation, STAT1 and STAT6 suppression, IL-10, IL-12 stimulation [85,86]
Methotrexate	Cervical, breast, lung, head and neck, lymacrophageoma, leukemia [134,135,136,137,138]	- Inhibits dihydrofolate reductase (DHFR) and nucleotide biosynthesis [139]	-Induces p53 activity in TAMs; contributes to the thymidylate synthase-dependent drug sensitivity [140]
Olaparib	Prostate, pancreatic, and breast cancer [141,142,143]	- Inhibits poly (ADP-ribose) polymerase (PARP) responsible for DNA break repair; - Leads to cancer cell apoptosis, especially during homologous recombination [144]	- Induces M1 polarization via IRF5 [145]
Radiotherapy, dose			
2 Gy	Rectal cancer [145]	- DNA double- and single-strand breaks; - ROS generation; - Activates cell repair following DNA damage [146,147]	- Activates IRF TF and up-regulates *TNFA*, *IFNG, IL6, IL8,* and *IL23* expression; - Induces M1 polarization via IRF5 activation
10 Gy	Colon carcinoma [148]	- Recruits ATM and activates Chk2 for DNA repair and checkpoint escape [146,147]	- Triggers RelB nuclear translocation and expression; - Stimulates Bcl-xL production and promotes TAM survival; - Stimulates IL-10, IL-6, CCL2 production and up-regulates HLA-DR, CD80, and CD86 cell membrane expression; - Induces M1 polarization
12–13.3 Gy	Melanoma [149] Lung cancer [150] Pancreatic ductal adenocarcinoma [151]	- Induces HMGB1 production by tumor and subsequent macrophage remodeling [152]	- Activates TFs STAT3, STAT6, SOCS3 and suppresses IRF5; - Stimulates CCL2, HDC, TGF-β, IL-6, IL-6R, IL-23, IL-13, Arg-1 and IL-12 and IFN-γ production; - Polarizes macrophages towards M2
20 Gy	Breast cancer [153]	- Induces tumor antigens and endogenous adjuvants production (heat shock proteins) and subsequent macrophage remodeling [154,155]	- Stimulates p50 activity; - Increases Arg-1 and IL-10 production; - Induces M2 polarization via p50 activation
1.5 Gy X-ray	Lung cancer [156]	- Decreases TGF-β production in the tumor microenvironment [157]	- Suppresses STAT6; - Promotes M1 polarization
66–70 Gy total in 2–2.2 Gy fractions	Head and neck cancers [158]	- Induces tumor-derived mitochondrial DNA production, TLR9 signaling, and macrophage remodeling [159]	- Implies STAT3 transcriptional activity; - Up-regulates *ARG1, LIF, TGFB1, IL4* and *IL5* expression in TAMs; - Induces M2 polarization
8 Gy	Pancreatic ductal adenocarcinoma [160]	- Stimulates TGF-β production in the tumor microenvironment [161]	- Activates STAT3; - Stimulates CCL2, CCL4, VEGF, and TGF-β production; - Induces M2 polarization
5–10 Gy proton irradiation	Lung adenocarcinoma [68]	- Induces ATM recruitment and DNA repair	- Promotes p65 nuclear translocation; - Induces M1 polarization

Legend and abbreviations used in Table 3. Treatment (column 1) strategies applied for majority of cancer types (column 2) induce principal alterations in cancer and tumor-associated cells impacting cell survival and metabolism (column 3). The therapeutic interventions additionally exhibit pro- or anti-inflammatory effects mediated by macrophages (column 4). Gy-Gray (Units); γ-gamma-irradiation; DNA-deoxyribonucleic acid; RNA-ribonucleic acid; Th2-T helper cell 2; ADP-ribose-adenosine diphosphate ribose; ROS-reactive oxygen species; ATM-ataxia telangiectasia mutated; Chk2-checkpoint kinase 2; HMGB1-high-mobility group protein B1; TLR9-toll-like receptor 9; JAK2-janus kinase 2; RelA, Bcl-xL-B-cell lymphoma-extra large; HLA-DR-human leukocyte antigen—DR isotype; SOCS3-suppressor of cytokine signaling 3; HDC-histidine decarboxylase; iNOS-inducible nitric oxide synthase; TNF-α-tumor necrosis factor alpha; TGF-β-transforming growth factor beta; Arg-1-arginase-1.

## Abbreviations

ADM	Adrenomedullin
Arg-1	Arginase-1
ATM	Ataxia telangiectasia mutated (serine/threonine kinase)
Bcl-xL	B-cell lymacrophageoma-extra large
CCL	Chemokine (C-C motif) ligand
CCR2	C-C chemokine receptor 2
CD40	Cluster of differentiation 40
CIITA	Class II, major histocompatibility complex, transactivator
COX2	Prostaglandin-endoperoxide synthase 2
CREB	cAMP response element-binding protein
CSF	Colony stimulating factor
CYP19A1	Cytochrome P450 Family 19 Subfamily A Member 1
CXCL1	The chemokine (C-X-C motif) ligand
C/EBP	CCAAT/enhancer binding protein
DHFR	Dihydrofolate reductase
EGF	Epidermal growth factor
ERK	Mitogen-activated protein kinase
eIF4E	Eukaryotic translation initiation factor
FN1	Fibronectin 1
Fizz1	Resistin-like beta
GBP6	Guanylate binding protein
GM-CSF	Granulocyte-macrophage colony-stimulating factor
Gy	Gray
GDF-15	Growth differentiation factor 15
HDL	High-density lipoprotein
HLA	Human leukocyte antigens
HMGB1	High-mobility group protein B1)
IFIT2	Interferon-induced protein with tetratricopeptide repeats 2
IFN	Interferon
IκB	Inhibitor of nuclear factor kappa B
IKK	IκB kinase
IL	Interleukin
iNOS	Inducible nitric oxide synthase
IRF	Interferon regulatory factor
JAK	Janus kinase
LPS	Lipopolysaccharide
L-Arg	L-arginine
LIF	Leukemia inhibitory factor
MafB	V-maf musculoaponeurotic fibrosarcoma oncogene homolog B
MHC	Major histocompatibility complex
MMP	Matrix metallopeptidase
Mnk	MAP kinase-interacting kinase
MYD88	Myeloid differentiation primary response 88
NK-kB	Nuclear factor kappa-light-chain-enhancer of activated B cells
NRF2	The nuclear factor erythroid 2-related factor 2
NSAIDs	Nonsteroidal anti-inflammatory drugs
PARP	poly-ADP-ribose polymerase
PDTC	Pyrrolidine dithiocarbamate
PD-L1	Programmed cell death 1
PGE2	Prostaglandin E2
PPAR	Peroxisome proliferator-activated receptors
RHD	Rel homology domain
ROS	Reactive oxygen species
SDF-1	Stromal cell-derived factor-1
STAT	Signal transducer and activator of transcription
TAM	Tumor associated macrophage
TF	Transcription factor
TGF-β	Transforming growth factor beta
TLR	Toll-like receptor
TNF-α	Tumor necrosis factor alpha
TRAF	TNF receptor associated factor
TRAM	TRIF-related adaptor molecule
HIF-1α	Hypoxia-inducible factor 1-alpha
VEGF	Vascular endothelial growth factor
Ym1	Chitinase 3-like 3
